# Multimodal imaging platform for enhanced tumor resection in neurosurgery: integrating hyperspectral and pCLE technologies

**DOI:** 10.1007/s11548-025-03340-1

**Published:** 2025-04-03

**Authors:** Alfie Roddan, Tobias Czempiel, Chi Xu, Haozheng Xu, Alistair Weld, Vadzim Chalau, Giulio Anichini, Daniel S. Elson, Stamatia Giannarou

**Affiliations:** The Hamlyn Centre for Robotic Surgery, Imperial College London, Exhibition Rd, London, SW7 2AZ UK

**Keywords:** Multimodal imaging, Neurosurgery, PCLE, Hyperspectral imaging, Platform

## Abstract

**Purpose:**

This work presents a novel multimodal imaging platform that integrates hyperspectral imaging (HSI) and probe-based confocal laser endomicroscopy (pCLE) for improved brain tumor identification during neurosurgery. By combining these two modalities, we aim to enhance surgical navigation, addressing the limitations of using each modality when used independently.

**Methods:**

We developed a multimodal imaging platform that integrates HSI and pCLE within an operating microscope setup using computer vision techniques. The system combines real-time, high-resolution HSI for macroscopic tissue analysis with pCLE for cellular-level imaging. The predictions of each modality made using Machine Learning methods are combined to improve tumor identification.

**Results:**

Our evaluation of the multimodal system revealed low spatial error, with minimal reprojection discrepancies, ensuring precise alignment between the HSI and pCLE. This combined imaging approach together with our multimodal tissue characterization algorithm significantly improves tumor identification, yielding higher Dice and Recall scores compared to using HSI or pCLE individually.

**Conclusion:**

Our multimodal imaging platform represents a crucial first step toward enhancing tumor identification by combining HSI and pCLE modalities for the first time. We highlight improvements in metrics such as the Dice score and Recall, underscoring the potential for further advancements in this area.

## Introduction

Neuro-oncological surgery presents unique challenges due to the need for high precision in tumor resection, which is the first step to initiate treatment and, if successful, can significantly improve the prognosis [1]. Several intra-axial tumors, especially gliomas, tend to infiltrate the brain parenchyma beyond the margins identified through conventional clinical imaging, a well-known problem in the neuro-oncological community [2]. The tumor-infiltrated areas are challenging to identify, and a generous resection of these equivocal regions is often problematic due to functionally active areas, which can cause a disability if damaged [3–5]. Meningiomas are slow-growing and invade the surrounding dura rather than the brain [6]. Still, they present similar challenges in extending the resection to the surrounding meningeal tissue or assessing the degree of brain invasion in the rare cases of more malignant sub-types [7]. Several preoperative and intra-operative supportive technologies have been introduced in clinical practice to address the problem of tumor resection, including neuronavigation [8], intra-operative ultrasound [9], fluorescence imaging [10, 11] and intra-operative MRI [12], to name a few. However, all these solutions show some limitations, either because of sub-optimal accuracy [13], operator-dependency [9], high costs or logistical issues [14].

Probe-based Confocal Laser Endomicroscopy (pCLE) is a high-resolution imaging tool that provides cellular-level visualization of tumor margins in vivo, aiding in more accurate tumor removal [15, 16]. While it enables detailed tissue examination and localized tumor characterization, its small field of view limits efficiency for larger areas, and the lack of tracking makes it easy for surgeons to lose track of scanned regions [17]. Hyperspectral imaging (HSI), on the other hand, provides information across a wide field of view. It is a noninvasive imaging technique that captures reflection data at wavelengths across the electromagnetic spectrum for each pixel [18]. HSI can be used to differentiate tissue types, identify abnormalities, and assist in disease diagnosis by analyzing the composition of specific tissues [19, 20]. Together, HSI and pCLE offer a unique blend of spectral and cellular morphology information, never before used for neurosurgical application. The combination provides a powerful, complementary solution whereby HSI offers rapid, broad-area detection while pCLE enables precise, cellular-level assessment for tumor identification.

In this work, we propose for the first time an innovative combination of HSI and pCLE for improved brain tumor removal. To achieve this: (1) we developed a novel platform that seamlessly integrates HSI and pCLE; (2) we tackle the surgical navigation challenges posed by pCLE alone and the complications that can arise when using HSI and pCLE without integration, enhancing usability and supporting surgeons in navigation; and (3) we demonstrate the diagnostic capabilities of this combined approach to effectively identify tumor regions by leveraging their complementary strengths using an ex vivo phantom study.

**Fig. 1:**
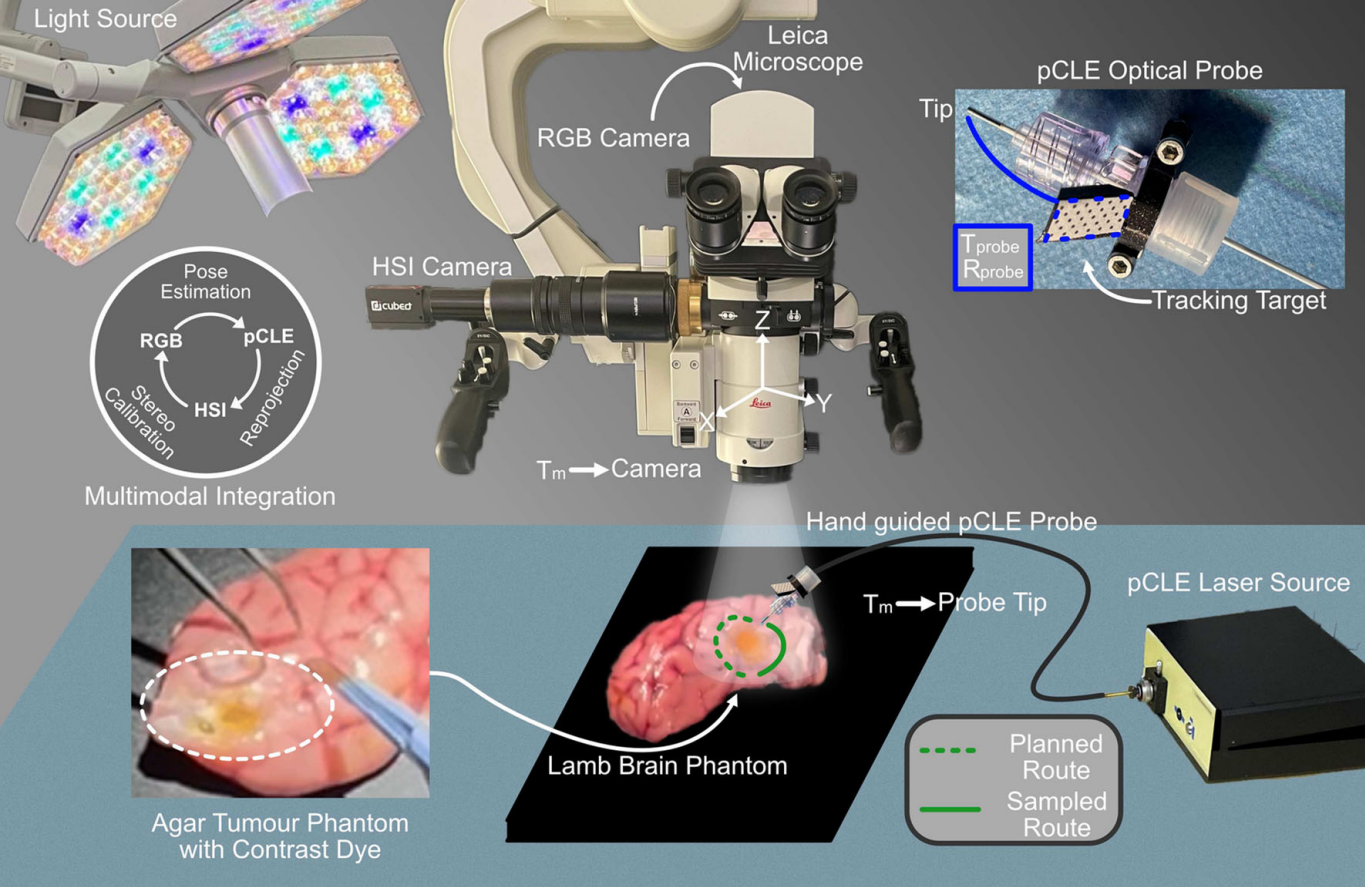
Our multimodal platform integrates RGB, HSI, pCLE. The lamb brain phantom was imaged macroscopically using HSI and RGB, both integrated into a Leica microscope. The pCLE, operated manually, performs microscale optical biopsies around the tumor margins. By tracking the pCLE, we can correlate its data with the HSI modality using a tracking target attached to the pCLE probe

## Multimodal imaging platform design

In our proposed method, we integrate the operating microscope with HSI and pCLE to create a multimodal platform for tissue analysis. An overview of our setup is depicted in Fig. 1. The operating microscope (LEICA M525 OH4) offers a magnified—although not truly microscopic—view of the surgical scene, and features an optical beam splitting system to direct light to multiple ports that share the same viewpoint. Therefore, the magnification and focal length controls which are integrated into the objective lens system have the same effect for the surgeon oculars and all attached imaging devices. We mounted a LEICA True Vision TZ+ 3D Surgical Camera on the rear binoculars, enabling 120 Hz white-light image capture, and this image provided the reference coordinate system. The output RGB images are composite 1920 $\times$ 1080 pixels, split down the middle between left and right camera. This setup allowed us to capture the surgical environment and detect the pCLE probe in real time.

For HSI, we attached a custom C-mount adapter to the optical port, allowing us to connect the CUBERT ULTRIS SR5 camera. This configuration allowed simultaneous white-light and hyperspectral image capture without disrupting the surgeon’s workflow. The CUBERT ULTRIS SR5 captures 51 spectral bands ranging from 450 to 850 nm with a 15 Hz frame rate with 275 $\times$ 290 pixel resolution. The measured illumination spectrum of the operating microscope’s light source reaches up to 770 nm, therefore we utilized 40 of the 51 spectral bands, restricting the range to 450–762 nm.

We integrated the HSI camera with a custom-designed pCLE system, featuring a 488 nm laser and a CMOS camera (Point Grey Flea3, Teledyne FLIR LLC, USA). The setup also includes a longpass fluorescence emission filter with a 500 nm cut-on wavelength (FEL0500, Thorlabs Inc., USA) and a laser line clean-up notch filter (NF488-15, Thorlabs Inc., USA) [21]. Additionally, the system is equipped with a high-resolution, flexible AQ-Flex19 fiber bundle probe from Cellvizio (Mauna Kea Technologies, France). This configuration allows for image capture at 120 frames per second, facilitating real-time, high-resolution cellular imaging with a $325~\upmu$m field of view, ideal for use during surgical procedures.

The pCLE system was positioned adjacent to the surgical setup, functioning independently from the operating microscope. The high frame rate and resolution of the RGB camera makes it ideal for precise pose estimation of the pCLE probe, which can then be projected onto the HSI image, ensuring accurate alignment between the modalities after calibration.

### Calibration of imaging modalities

Each modality requires calibration to ensure consistent data across multiple acquisitions. The operating microscope is our reference frame, with high-resolution stereo data provided by the RGB camera. To ensure consistent image color, we performed a white-balance calibration to correct for variations in lighting from the operating microscope’s light source. This step guarantees consistent and reliable color data with good contrast.

Similar to the RGB white-balance calibration, the HSI modality also requires a calibration step due to the varying lighting conditions. To ensure hyperspectral data remains unaffected by these variations, images were captured with a reflectance target under standard lighting (white reference) and also while the sensor was covered (dark reference), both necessary for white-dark calibration. White-dark calibration subtracts the dark reference image ($D$) from the raw image ($R$), which was then normalized by dividing pixel-wise by the white reference image ($W$) minus the dark reference, i.e., $\text {Calibrated Image} = \frac{R - D}{W - D}$. The outcome is a hyperspectral image represented in units of reflectance fraction.

For the pCLE system, background subtraction was performed by capturing a reference image without fluorescence, which was subtracted from each subsequent image. This removed interference from ambient light, including the surgical microscope, ensuring consistent and accurate imaging focused on the fluorescent signal.

### Spatial alignment of multimodal data

**Fig. 2:**
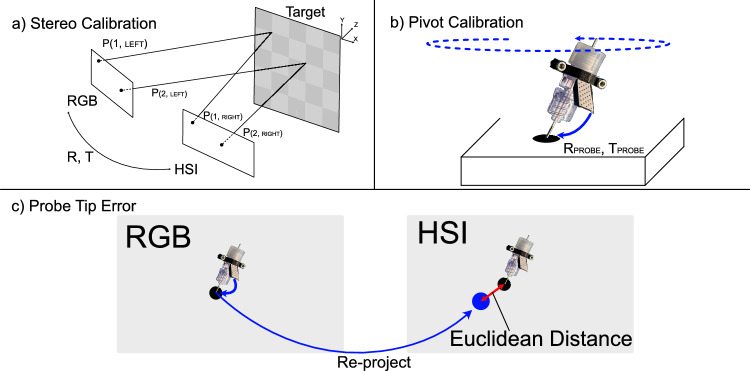
Calibration methodology and reprojection error analysis: **a** Stereo calibration between RGB camera and HSI camera, where R and T are the rotation and translation between left and right cameras (found during calibration). **b** Demonstration of the pivot-based calibration process showing alignment points between imaging systems. $R_{probe}$ and $T_{probe}$ are the rotation and translation between the marker and the probe tip to be found during the calibration process. **c** Visualization of the reprojection error calculation, illustrating the spatial discrepancy between corresponding points in the RGB and hyperspectral image data

To align the different sensor data into a joint coordinate frame, we performed a spatial calibration across the entire platform. Our two primary macro imaging modalities are the left hand image from the surgical microscope and the HSI. To transfer information between these two modalities, we performed stereo calibration using 30 images of a checkerboard pattern, a depiction of this is shown in Fig. 2a. These images allow us to estimate the intrinsic parameters of each camera individually ($K_\textrm{left}$, $K_\textrm{right}$), as well as the extrinsic parameters (*T* and *R*) between them. Given a world point in the left camera coordinate frame, $P_\textrm{left}$, we can estimate the same point in the right coordinate frame, $P_\textrm{right}$ as $P_\textrm{right} = R \cdot P_\textrm{left} + T.$ To then project $P_\textrm{right}$ onto the hyperspectral camera’s image plane we can use:

1 $$\begin{aligned} \begin{bmatrix} u_\textrm{right} \\ v_\textrm{right} \\ 1\end{bmatrix} = K_\textrm{right} \cdot \frac{1}{Z_\textrm{right}} \cdot P_\mathrm{\mathrm right}, P_{right} = \begin{bmatrix} X_\textrm{right} \\ Y_\textrm{right} \\ Z_\textrm{right}\end{bmatrix} \end{aligned}$$

where, $(u_\textrm{right}, v_\textrm{right})$ represents the pixel location on the right image.

To map the position of optical biopsy sites captured by the pCLE probe onto the combined HSI and color camera data, we need to localize the probe tip. A simple keydot marker was attached to the probe, allowing it to be detected in the color image. We used OpenCV [22] for marker detection, followed by pose estimation using the RANSAC perspective-n-point algorithm, which provided the 6 degrees of freedom pose of the marker. To accurately locate the biopsy site at the probe tip, we begin by estimating the translation and rotation from the keydot to the tip. As the probe remains fixed in place, the translations ($T_{\text {probe}}$) and rotations ($R_{\text {probe}}$) were estimated using pivot calibration [23], as depicted in Fig. 2b. This method involves keeping the probe tip stationary while capturing multiple images as the probe body rotates through different orientations, a depiction of this process is shown in Fig. 2a. Since the tip position remains constant in 3D space while the marker position varies, a system of equations can be formulated to solve for the rigid transformation between the tracked marker and the probe tip. Using this transformation, the probe tip position $P_{\text {probe}}$ can be determined from the marker’s world coordinates $P_{\text {marker}}$:

2 $$\begin{aligned} P_{\text {probe}} = R_{\text {probe}} \cdot P_{\text {marker}} + T_{\text {probe}} \end{aligned}$$

This approach ensures accurate tracking of the probe tip relative to the marker. To map the optical biopsy sites on the image plane of the hyperspectral camera, Eq. (1) can be applied on the 3D position of the tip of the pCLE probe.

To assess probe tip localization accuracy, we developed a systematic evaluation protocol that measures "probe tip error" - the reprojection error between the estimated probe tip pose in the RGB camera and its corresponding 2D pixel location in the HSI. First, we captured images of the probe touching multiple reference markers visible in both cameras. Since the precise pixel coordinates of each reference marker’s center were known in the right camera’s image plane, these served as our ground truth locations. For each position, we performed pose estimation of the keydot marker to obtain its orientation and position, then calculated the 3D position of the probe tip using Eq. (2). This 3D world point was subsequently reprojected onto the image plane of the hyperspectral camera. The probe tip error was quantified by calculating the Euclidean distance between the projected pixel location and the known center of the reference marker being touched. A depiction of this protocol is shown in Fig. 2c. By averaging these measurements across multiple reference markers, we established a robust metric for our system’s spatial accuracy. This approach, similar to the method in [24], provided a quantitative assessment of both the geometric transformation reliability and the overall precision of our multi-camera tracking system. The current clinical recommendation for tumor resection margins in gliomas range from 0.5 to 3.0 cm beyond the gadolinium-enhancing region of the tumor [25–27]. Therefore, we consider a probe tip error below 1 mm to be reasonable for accurate tumor identification, ensuring the system’s reliability in clinical applications.

## Ex vivo dataset

**Table 1:** This table presents the volume of injected agar-acriflavine and the size (height and width) of the exposed tumor at the selected key frame

Sample #	Volume (ml)	Size (mm)	Spectra			pCLE images	
			BG	Healthy	Tumor	Healthy	Tumor
1	2	15.8 $\times$ 17.6	50176	28202	1372	614	852
2	1	6.2 $\times$ 5.0	53811	25723	216	484	214
3	2	13.6 $\times$ 11.8	43725	34841	1184	572	192
4	2	14.0 $\times$ 12.1	53941	24477	1332	218	563
5	1	7.1 $\times$ 8.7	53827	25427	496	319	262

The last 5 columns detail the class distribution of HSI spectra and pCLE images across the dataset

We designed a realistic ex vivo brain model to test our multimodal platform. Following the experimental setup of [28], we employed a lamb brain phantom due to its close resemblance to human brain anatomy [29–31]. Despite minor spectral deviations, the structural similarity ensures comparable responses properties from HSI and pCLE for non-fluorescent tissue, validating its effectiveness for our study. Additionally, the lamb brain model is cost-efficient and readily available, making it an ideal choice for this application.

In order to test our multimodal platform for tumor identification, we then created artificial tumor masses within the lamb brains. After reviewing various solutions for tumor simulation reported in the literature, including plastic biomodels [32], 3D printing [33], polyvinyl alcohol [34], and several hydrogels [35, 36], we decided on using an injectable agar tumor mass model [28]. The agar was selected as it closely simulates the texture and optical light diffusion of brain tumors [37, 38]. Being injectable, it can create phantom subcortical masses without external disruption, simulating trans-cortical approaches with pre- and post-exposure acquisitions [28]. As well as being quick to prepare and cost-effective, it was also possible to add acriflavine, the contrast agent required for the pCLE imaging modality, which would be difficult to do for the other tumor phantom materials mentioned above. The addition of acriflavine enables clear differentiation between agar and non-agar regions through pCLE imaging, where areas containing agar (representing tumor tissue) produce a bright fluorescent response, creating an ideal simulation of tumor visualization in a clinical scenario.

Our dataset is comprised of five lamb brain models. Each lamb brain model was approximately 10 $\times$ 7 $\times$ 3.5 cm in size, and we kept the microscope at a distance of 30 cm from the specimen. At this height, the pixel size is 0.12 mm for the RGB camera and 0.31 mm for the hyperspectral camera, which is more prominent due to the smaller resolution of the hyperspectral camera. We created a 20% agar solution [28] and added acriflavine at a ratio of approximately 2:1 (agar to acriflavine) to ensure a high enough concentration of fluorescent dye to be seen by the pCLE system. This mixture was then injected into each lamb brain model, volumes ranged from 1 to 2 ml and allowed to set for 10 min. With this phantom setup, we are able to consistently simulate tumor-like masses in our brain phantoms, which are discriminable in all three modalities available in our platform: RGB (white light), HSI, and pCLE.

The image acquisition protocol began with surgical exposure of the tumor core in the lamb brain model, performed by a neurosurgeon who removed the overlying tissue. While tumor localization was guided by the injection site in this phantom study, clinical applications would utilize preoperative imaging for initial targeting. Following exposure, we used HSI for macro-level tumor localization and pCLE for subsequent detailed margin assessment. For pCLE, we established a comprehensive sampling approach that included three distinct regions: healthy brain tissue (sampled from areas clearly separate from the tumor), tumor core, and tumor margins. Margin assessment involved 15–20 discrete sampling points per specimen.

We created RGB annotations of the tumor region based on the known injection parameters and visible color differentiation between agar and brain tissue. These annotations were subsequently transferred to the HSI domain through RGB-HSI co-registration. To establish pCLE ground truth, we defined our binary classification using pCLE images from healthy regions and tumor core, while margin scans were reserved exclusively for testing, ensuring clear delineation between tissue types during training and preventing data contamination in our evaluation protocol. Our three classes are healthy brain tissue (healthy), tumor agar-acriflavine mixtures (tumor) and background (BG) as non-tissue. In the RGB annotations, healthy brain tissue is represented in green, tumor regions are depicted in red, and the background is shown in black. The dataset is summarized in Table 1.

**Fig. 3:**
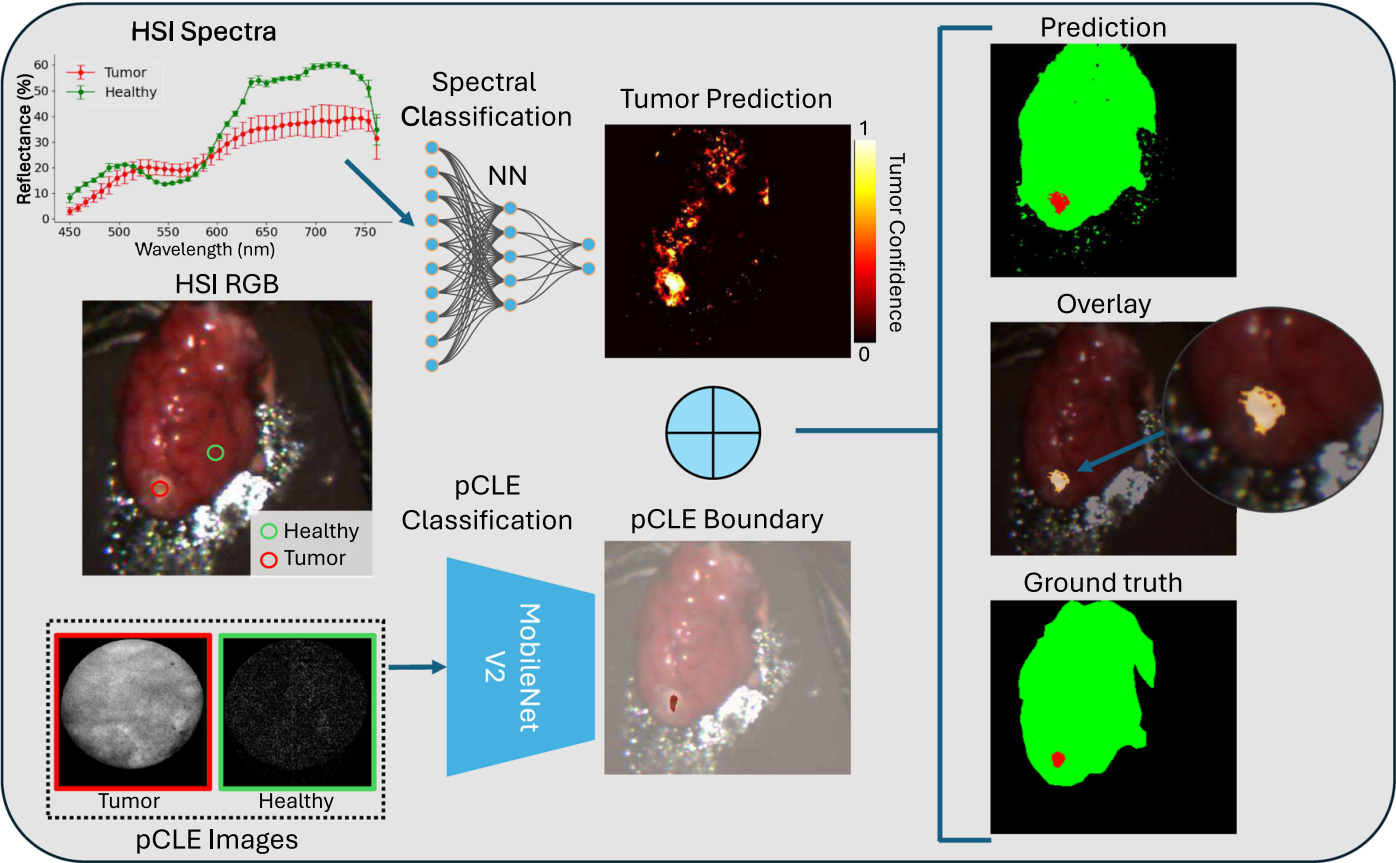
Overview of multimodal tumor identification algorithm. We show a healthy (green) and tumor (red) case with the HSI modality in the top and pCLE in the bottom of the image. The combination of the two modalities was done with a logical OR operation and a subsequent component filtering step

**Table 2:** Spatial and timing statistics of our multimodal platform

Reprojection error (pixels) $\downarrow$	Probe tip error (pixels) $\downarrow$	pCLE scan time (s) $\downarrow$
0.34 $\pm 0.1$	$1.2\pm 0.2$	$9.6 \pm 4.1$

**Table 3:** HSI spectral classification results showing Dice for individual classes and average (Avg)

Dice $\uparrow$					
Model	BG	Healthy	Tumor	Avg	FPS $\uparrow$
SVM	$98.0 \pm 1.4$	$95.7 \pm 1.8$	$55.5 \pm 17.6$	$83.0 \pm 6.9$	0.014
NN	$98.2 \pm 0.9$	$94.7 \pm 2.1$	$53.1 \pm 15.0$	$82.0 \pm 5.7$	1.47

Additionally the frames per second (FPS) for SVM and Neural Network (NN) model is shown

**Table 4:** pCLE results for binary classification of tumor showing macro accuracy and F1

Model	Accuracy $\uparrow$	F1 $\uparrow$	FPS $\uparrow$
EfficientNet	95.7 $\pm 2.3$	95.2 $\pm 2.1$	39.1 $\pm 2.8$
MobileNetV2	$95.6 \pm 2.0$	95.1 $\pm 1.8$	220.5 $\pm 19.5$

Additionally the frames per second (FPS) is shown

**Table 5:** Results overview for the multimodality algorithm showing Dice and Recall

	BG	Healthy	Tumor	Avg
*Dice* $\uparrow$				
HSI	$98.2 \pm 0.9$	$94.7 \pm 2.1$	$53.1 \pm 15.0$	$82.0 \pm 5.7$
pCLE	–	–	$70.5 \pm 5.0$	–
HSI LCC	$98.2 \pm 0.9$	$95.2 \pm 1.9$	$52.6 \pm 30.5$	$82.0 \pm 11.1$
HSI+pCLE	$98.2 \pm 0.9$	$95.7 \pm 0.7$	$71.7 \pm 2.0$	$88.5 \pm 0.7$
*Recall* $\uparrow$				
HSI	$97.3 \pm 1.6$	$94.5 \pm 2.9$	$82.8 \pm 8.3$	$91.5 \pm 3.1$
pCLE	–	–	$68.8 \pm 12.7$	–
HSI LCC	$97.3 \pm 1.6$	$96.1 \pm 1.8$	$61.9 \pm 36.7$	$85.1 \pm 13.2$
HSI+pCLE	$97.3 \pm 1.6$	$96.1 \pm 1.5$	$94.6 \pm 0.9$	$96.0 \pm 0.7$

Individual modalities and the combination are shown. BG stands for background, and Avg represents the average performance across all classes

## Multimodal tumor identification

In this section, we describe the design of our multimodal tumor identification algorithm consisting of HSI, pCLE and the combination of both. To roughly evaluate our model under low data regime we applied 5-fold cross-validation where we always have 4 of the samples for training and evaluation and one for testing. To address class imbalance, we applied an undersampling approach by reducing the majority class size based on annotation statistics, randomly selecting a subset of instances to match or be closer in size to the minority class. For the HSI segmentation approaches we opted for pixel-wise methods, in this low data regime. The two top-performing spectral classification methods from [39] namely, SVM [40] and a neural network are selected. The SVM was trained with a regularization parameter equal to 1 and a radial basis kernel. The neural network, on the other hand, is a three-layer architecture with hidden layer sizes of 128 and 64, followed by a final output layer. Training employed cross-entropy loss and the Adam optimizer [41] with a learning rate of 0.001. In recent years, machine learning has been the dominant method for pCLE classification [42, 43]. Our study prioritized optimizing inference speed, as the model needs to classify and provide results in real-time, so we evaluated two convolutional neural networks known for their fast inference: MobileNetV2 and EfficientNet [44, 45]. The pCLE models were trained with the same loss, optimizer and learning rate as the above HSI neural network model. All models compatible with GPU acceleration were trained using PyTorch [46] on an NVIDIA 3090 GPU. For the SVM, we utilized the Scikit-learn implementation [47].

To combine the above imaging modalities, we use a non-machine learning approach that enhances interpretability and simplifies use in surgical environments. For the HSI+pCLE we first computed the logical OR between the two modalities, highlighting regions where either modality identified potential tumor areas. Since pCLE focuses directly on the tumor, we refined this by retaining only the largest connected component (LCC) in the HSI segmentation that contains the pCLE points, removing other segments resulting in a pCLE-informed HSI segmentation. The filtered out tumor regions are replaced by the next highest confidence score from the HSI models. This entire pipeline is depicted in Fig. 3 For HSI-only segmentation, without pCLE data, we included an additional comparison by retaining only the largest component in HSI and removing all smaller segments (HSI LCC) which is a similar filtering approach but without the informed pCLE information.

This study design allows us to evaluate the performance of each modality individually and in combination, ensuring that the resulting algorithm is both robust and practical for surgical applications, even in data-limited settings.

## Results

For our multimodal imaging platform, the precise integration of different modalities is crucial to avoid errors from spatial misalignment. To assess the quality of our integration, we measured the reprojection error from HSI to RGB through stereo calibration, as outlined in Sect. 2, achieving a reprojection error as low as 0.34 pixels. We also evaluated the probe tip error, finding it to be 1.2 pixels with a standard deviation of 0.2 pixels. These low reprojection errors ensure spatial consistency across modalities. Finally, we measured the average time required to scan the whole tumor margins with the pCLE probe, which was 9.6 s with a standard deviation of 4.1 s. The results are summarized in Table 2.

Using our previous estimation of pixel size from Sect. 3, with a probe tip error of 1.2 pixels, this corresponds to a registration error of $\sim 0.14$ mm. As mentioned in Sect. 3 our target was sub-millimeter error. This ensures precise margin evaluation and reliable tumor classification, as the error is significantly smaller than the structures being measured.

We evaluated the two HSI models-SVM and NN-introduced in Sect. 4. As shown in Table 3, both models achieved similar Dice scores, aligning with findings in the literature, but the neural network demonstrated nearly ten times faster inference speed, making it the preferred choice for spectral classification to meet the real-time demands of surgery. Given the significant speed advantage, we selected the NN model for our combined algorithm. Additionally, we evaluated the pCLE modality, as presented in Table 4. Both models-EfficientNet and MobileNetV2-showed comparable classification performance. However, MobileNetV2 was approximately six times faster, leading to its selection for the combined algorithm.

The combined algorithm was evaluated in its individual parts as detailed in Sect. 4. The results presented in Table 5 demonstrate the performance of the different models in terms of Dice and Recall, with a specific focus on tumor segmentation performance. The HSI model alone shows a promising average Dice score (Avg) and good recall for the tumor class, indicating its effectiveness in capturing tumor regions. In comparison, the pCLE model achieves a higher Dice score for the tumor class, but suffers from a notable drop in recall, leading to unidentified tumor regions. As the pCLE is a probe-based approach we can only use it for the tumor class because it is not feasible to sample every single pixel in the image. To reduce the false positives and therefore increase the Dice of the HSI for the tumor class we used the largest component of the HSI only to remove overpredicted areas. The almost twice as high standard deviation of this model compared to the HSI-only model indicates that the largest predicted tumor area might not correspond to the ground truth. Therefore we see no improvements on the metrics.

The combination of HSI+pCLE, demonstrates significantly improved Dice for the tumor class compared to HSI only (+ 18.6), and most importantly, much higher tumor recall compared to pCLE only (+ 25.8). This improvement highlights the value of combining modalities to enhance tumor localization and ensure that critical tumor areas are not missed during segmentation.

## Discussion and conclusion

In this work, we developed a multimodal imaging platform combining HSI and pCLE, marking an important first step toward improving tumor localization during simulated surgical procedures. By integrating both modalities, we show that the system provides clinically valuable insights, highlighting the potential for further exploration in this area. We observed that pCLE has the potential to refine tumor margins, yet the system lacks a feedback loop where the HSI prediction probability could guide surgeons to critical sampling points. Integrating such a mechanism could significantly enhance tumor margin identification further, which is essential for clinical applications.

Future efforts will prioritize the implementation of markerless pose estimation for the pCLE probe, simplifying the system while ensuring precise pose tracking. Furthermore, to prepare for real surgical applications, we plan to expand the dataset to include hundreds of samples. This larger dataset will facilitate the refinement of our algorithms and lead to substantial improvements in tumor boundary detection, ensuring the system’s robustness and reliability in clinical settings. We also plan to develop an interactive approach where HSI informs the surgeon about regions to sample with pCLE, further optimizing tumor margin refinement and enhancing the clinical utility of the system.
